# The Hippo Pathway in Breast Cancer: The Extracellular Matrix and Hypoxia

**DOI:** 10.3390/ijms252312868

**Published:** 2024-11-29

**Authors:** Hanyu Yang, Jiaxin Yang, Xiang Zheng, Tianshun Chen, Ranqi Zhang, Rui Chen, Tingting Cao, Fancai Zeng, Qiuyu Liu

**Affiliations:** 1 School of Pharmacy, Southwest Medical University, Luzhou 646000, China; 2 Laboratory of Biochemistry and Molecular Biology, School of Basic Medical Science, Southwest Medical University, Luzhou 646000, China; 3 School of Basic Medical Science, Southwest Medical University, Luzhou 646000, China; 4 Leiden Academic Centre for Drug Research, Leiden University, 2333 CC Leiden, The Netherlands

**Keywords:** breast cancer, hippo signaling pathway, hypoxia, extracellular matrix (ECM), tumor microenvironment (TME)

## Abstract

As one of the most prevalent malignant neoplasms among women globally, the optimization of therapeutic strategies for breast cancer has perpetually been a research hotspot. The tumor microenvironment (TME) is of paramount importance in the progression of breast cancer, among which the extracellular matrix (ECM) and hypoxia are two crucial factors. The alterations of these two factors are predominantly regulated by the Hippo signaling pathway, which promotes tumor invasiveness, metastasis, therapeutic resistance, and susceptibility. Hence, this review focuses on the Hippo pathway in breast cancer, specifically, how the ECM and hypoxia impact the biological traits and therapeutic responses of breast cancer. Moreover, the role of miRNAs in modulating ECM constituents was investigated, and hsa-miR-33b-3p was identified as a potential therapeutic target for breast cancer. The review provides theoretical foundations and potential therapeutic direction for clinical treatment strategies in breast cancer, with the aspiration of attaining more precise and effective treatment alternatives in the future.

## 1. Introduction

Breast cancer, as one of the most common malignant tumors in women worldwide, continues to have high incidence and mortality rates, posing a significant threat to women’s health. It is crucial to investigate the biological characteristics of tumor cells to enhance treatment efficacy and improve patient prognosis. The tumor microenvironment (TME) plays a vital role in the occurrence, progression, therapeutic response, and prognosis evaluation of breast cancer. The extracellular matrix (ECM) is primarily composed of macromolecules such as collagen, laminin, elastin, proteoglycans, and various cytokines, forming a complex network among cells. These molecules not only provide essential three-dimensional structural support for cells but also regulate critical biological functions such as cell proliferation, migration, and differentiation, playing an important role in maintaining tissue architecture and physiological responses [1]. Concurrently, hypoxia in the TME is widely recognized as a hallmark of solid tumors and significantly impacts tumor growth, invasive potential, therapeutic response, and immune evasion [2]. The development of a hypoxic microenvironment usually arises from rapid tumor cell proliferation coupled with insufficient angiogenesis, resulting in inadequate blood supply. Under hypoxic conditions, tumor cells adapt through various mechanisms and drive tumor progression. Both the ECM and hypoxia collaboratively influence the tumor cell behaviors and are thought to be closely related to tumor metastasis and treatment resistance [3].

It is essential to consider the combined effects of ECM and hypoxia when exploring the biological characteristics of tumor cells. The ECM provides physical support to tumor cells and participates in regulating cellular signaling processes through interactions with receptors on the cell surface. These interactions influence cell proliferation, migration, differentiation, and apoptosis [4,5]. As a key stressor in the TME, hypoxia profoundly alters cellular metabolic states and gene expression profiles. Under hypoxic conditions, tumor cells adapt to hypoxia through activation of hypoxia-inducible factors (HIFs) and other transcription factors that regulate the expression of a range of genes, thereby promoting tumor cell survival, angiogenesis, and metabolic reprogramming [2,6].

Under hypoxic conditions, the ECM undergoes significant changes in both composition and structure, which are critical for tumor cell adaptation and cancer progression. Hypoxia can modify ECM composition and structure by enhancing the ability of cells to adapt to low-oxygen environments and potentially promoting the development of more malignant tumor phenotypes [7]. Collagen, one of the most abundant proteins in the ECM, provides essential structural support and mechanical strength to tissues [8]. The deposition and cross-linking of type I collagen are increased, which enhances the interaction between tumor cells and the ECM, thereby promoting the invasive and migratory capabilities of tumor cells [9]. Hypoxia-induced collagen deposition and cross-linking can make the ECM more rigid, and such alterations in stiffness can affect cellular behaviors, including changes in cell morphology, increased migratory ability, and elevated proliferation activity. Studies have shown that tumor cells often exhibit greater invasiveness on stiffer ECM. Under hypoxia, tumor and stromal cells (such as cancer-associated fibroblasts) can secrete matrix metalloproteinases (MMPs) and other proteases that degrade and remodel the ECM [10]. Additionally, these ECM alterations participate in cell signaling by interacting with receptors like integrins on the cell surface, which regulate cell survival, proliferation, and differentiation [11]. Changes in ECM composition and structure under hypoxic conditions may enhance cellular adaptation to hypoxia. Moreover, hypoxia may influence ECM synthesis and degradation by activating signaling pathways, such as the HIF-1α pathway, further modifying the ECM composition and structure [12].

Changes in the ECM have profound impacts on the hypoxic environment. The physical properties of the ECM, particularly its stiffness, significantly affect cellular behavior [11,13]. An increase in ECM rigidity can enhance glycolytic activity in cells through the activation of integrin-mediated signaling pathways, thereby exacerbating hypoxic conditions [9,13]. The metabolic reprogramming can further impact tumor progression and therapeutic responses by promoting tumor cell adaptation to hypoxia [10]. The structure and chemical properties of the ECM are crucial for new blood vessel formation. Under normal circumstances, the ECM provides necessary structural support and signaling to facilitate the migration of endothelial cells and the formation of lumens [10]. However, abnormal ECM deposition and remodeling disrupt normal angiogenesis, leading to chaotic vascular networks [8,14]. This disorganization not only impairs effective oxygen transport but also results in severe hypoxia, as the distance between blood vessels exceeds the effective range for oxygen diffusion [15]. Hypoxia further stimulates the expression of angiogenic factors such as VEGF to compensate for inadequate oxygen supply, leading to the formation of more abnormal blood vessels and further exacerbating hypoxia [14,16].

In particular, the Hippo signaling pathway may exhibit significant changes in its function and regulatory mechanisms under the dual influence of the ECM and hypoxia. The Hippo signaling pathway, as a critical signaling network in cellular biology, regulates organ size and shape by controlling cell proliferation, apoptosis, migration, and tissue regeneration. The activation of MST1/2 is considered the starting point of the Hippo pathway. Once activated, MST1/2 further activates LATS1/2, which phosphorylates and inhibits the functions of the two main effectors: Yes-associated protein (YAP) and transcriptional co-activator with PDZ-binding motif (TAZ). Activation of the Hippo signaling pathway results in the phosphorylation of YAP and TAZ, leading to their binding with 14-3-3 proteins, which prevents their translocation into the nucleus and suppresses the expression of downstream genes. Conversely, when the signaling pathway is inhibited, YAP and TAZ translocate to the nucleus, where they interact with the TEAD family of transcription factors to activate genes that promote cell proliferation and inhibit apoptosis, processes that are closely linked to tumorigenesis. Under normal physiological conditions, when the Hippo pathway is activated, YAP/TAZ are phosphorylated and sequestered in the cytoplasm, inhibiting their transcriptional activity. However, changes in the ECM and hypoxic conditions within the TME may disrupt this process [17,18]. Consequently, a comprehensive understanding of the mechanisms of the Hippo signaling pathway in breast cancer, particularly under the dual influences of ECM and hypoxia, is crucial for identifying new tumor targets and developing effective treatment strategies.

The purpose of this review is to summarize the complicated crosstalk between the ECM and the hypoxic microenvironment mediated by the Hippo signaling pathway in breast cancer and to explore how these interactions shape the biological characteristics of breast cancer. The review article begins with an overview of the mechanisms of the Hippo signaling pathway, followed by a detailed elaboration on how the ECM and hypoxic conditions influence breast cancer cell behavior through the Hippo signaling pathway. Moreover, we investigated the role of microRNAs (miRNAs) in regulating ECM components and evaluated their potential as therapeutic targets. This review provides insights and basic information on molecular mechanisms for clinical treatment in breast cancer patients in the future.

## 2. Overview of Hippo Signaling Pathway 

The Hippo signaling pathway is an intracellular signaling mechanism that widely exists in many organisms, first discovered in *Drosophila*, where it serves as a key factor coordinating the balance between cell proliferation and apoptosis, precisely regulating organ size and shape [19]. Central to this pathway is a series of cascading reactions initiated by the Hippo (Hpo) kinase, which binds to its co-factor Salvador (Sav) to activate the downstream Warts (Wts) kinase interacting with Mob-as-tumor-suppressor (Mats) to achieve full activation [20,21]. The pathway effector, Yorkie (Yki), can enter the nucleus and bind to the transcriptional co-factor Scalloped (Sd) to activate a series of genes that promote cell growth and division when it is not phosphorylated [22]. When the Hippo signaling pathway is activated, Yki is phosphorylated by Wts, leading to its retention in the cytoplasm and preventing it from entering the nucleus to exert its functions, thus inhibiting cell proliferation and promoting apoptosis. The finely tuned regulatory mechanism ensures appropriate control of cell numbers during development, preventing excessive organ growth or underdevelopment, which is crucial for maintaining normal tissue structure and function [20,22].

Due to its high conservation, the Hippo signaling pathway is also present in humans, with core components and functions that are fundamentally similar to those in *Drosophila*, but it incorporates a more complex regulatory network and feedback mechanisms [23]. When the Hippo pathway is not activated, YAP and TAZ are predominantly located in the cytoplasm, where they can enter the nucleus via nuclear localization signals and bind to transcriptional factors, ultimately promoting the expression of downstream genes. However, when cells come into contact with each other, adhere to the ECM, or experience stress and damage [24], kinases such as MST1/2 (homologs of Hippo) and LATS1/2 (homologs of Warts) will be activated by a series of upstream signals, triggering a cascade of reactions within the cell that regulates the activity of the transcriptional co-factors YAP/TAZ [25,26]. Specifically, YAP/TAZ are phosphorylated by LATS1/2, leading to their binding with 14-3-3 proteins, which masks the nuclear localization sequences of YAP/TAZ, preventing them from entering the nucleus to perform their functions, causing them to accumulate in the cytoplasm, and ultimately resulting in degradation.

### 2.1. Initiation and Regulation of the MST-LATS Kinase Cascade

The MST-LATS kinase cascade is the core of the Hippo signaling pathway and is regulated by multiple upstream signals [24,27]. The regulatory process typically begins with the maintenance of cell polarity and the perception of cell-cell contact [28]. Cell polarity complexes, including CRUMBS, PAR, and SCRIB [29], work synergistically with tight junction proteins such as E-cadherin and α-catenin to monitor cell density and contact [29,30], influencing the overall activity of the pathway [31]. The PAR complex, primarily composed of PAR3, PAR6, and aPKC, is activated by asymmetric signals on the cell membrane. Once activated, the PAR complex allows Par3 to interact with KIBRA, inhibiting the Hippo signaling pathway and promoting the nuclear translocation of YAP/TAZ, which in turn activates the expression of downstream genes, facilitating cell proliferation and survival [32].

G protein-coupled receptors (GPCRs) are a large family of transmembrane receptors that can sense and respond to a variety of extracellular signals, which can also influence the Hippo pathway. The signals sensed by GPCRs are mediated through G proteins, which can either positively or negatively regulate the overall activity of the Hippo signaling pathway [33,34]. Upon activation, some GPCRs engage G protein subunits (usually from the Gαq/11 subfamily) to activate phospholipase C (PLC), leading to the production of inositol trisphosphate (IP3) and diacylglycerol (DAG). IP3 promotes the release of calcium ions from the endoplasmic reticulum into the cytoplasm, increasing intracellular calcium concentration. The elevated calcium concentration can activate a series of downstream effector molecules, such as calcium/calmodulin-dependent kinases (CaMKs) and other calcium-sensitive kinases, which can further activate upstream kinases in the Hippo pathway, such as MST1/2, promoting the activation of the Hippo signaling pathway [35]. Conversely, certain GPCRs can inhibit adenylate cyclase (AC) through G protein subunits (such as the Gαi/o subfamily), reducing the production of cAMP and subsequently inhibiting protein kinase A (PKA) activity. Because PKA typically acts as a negative regulator of the Hippo signaling pathway, its increased activity leads to the inhibition of the Hippo pathway, resulting in decreased phosphorylation levels of YAP/TAZ, which allows YAP/TAZ to enter the nucleus and activate the expression of downstream genes, promoting cell proliferation [36].

### 2.2. SAV1-Mediated Formation of Active MST1/2 Dimers

Salvador Family WW Domain Containing 1 (SAV1) plays a pivotal role in cell cycle regulation, apoptosis, and DNA damage response in the Hippo signaling pathway by modulating the activity of MST1/2 kinases. When MST1/2 is phosphorylated by upstream signals, SAV1 binds to them. As a crucial adaptor protein in the Hippo pathway, SAV1 forms a complex with MST1/2, facilitating their dimerization, which is generally regarded as the initial step in activating the Hippo signaling pathway [37]. The kinase activity of MST1/2 relies on the dimeric structure; in the absence of activating signals, MST1/2 typically exists as inactive monomers. Interactions between MST1/2 molecules are induced upon binding with SAV1, promoting the formation of stable dimers [37,38], the structural transition of which is essential for activating MST1/2 kinase activity, as dimeric MST1/2 is more susceptible to autophosphorylation, leading to its activation [37]. Additionally, SAV1 serves as a molecular scaffold that correctly aligns the active sites of MST1/2 kinases for autophosphorylation [39]. In the MST1/2 dimer, the active site of one molecule can phosphorylate specific serine or threonine residues on the other molecule. The autophosphorylation process is a critical step in MST1/2 activation. The presence of SAV1 optimizes the spatial arrangement between MST1/2 molecules, enhancing the efficiency of autophosphorylation and accelerating MST1/2 activation [37,39].

### 2.3. Dynamic Regulation of SAV1-MST1/2 Interaction

It is noteworthy that the interaction between SAV1 and MST1/2 is dynamically regulated by intracellular and extracellular signals, protein modification states, and interactions with other signaling molecules [40]. This multi-layered regulatory mechanism ensures that the SAV1-MST1/2 complex can respond to diverse physiological and pathological conditions, thereby playing an important role in intracellular signaling functions.

In multicellular organisms, cell-cell contact and tissue density are critical factors influencing cell survival and functional changes. Generally, the rapid proliferation of tumor cells leads to an increase in cell density [41]. When cell density reaches a certain threshold, specific proteins on the cell membrane, such as NF2/Merlin, can sense this change and become activated [42,43]. NF2/Merlin contains a FERM (Four-point-one, Ezrin, Radixin, Moesin) domain, which primarily facilitates interactions with other proteins by recognizing conserved amino acid sequences like PPxY (Pro-Pro-x-Tyr) and DXV (Asp-any-Val). The molecular structure of MST1/2 includes interaction sites for NF2/Merlin, enabling NF2/Merlin to recognize and bind to MST1/2, promoting its association with SAV1 [44]. Moreover, evidence suggests that the active state of NF2/Merlin significantly influences the dynamic remodeling of actin filaments. NF2/Merlin inhibits the aggregation of actin filaments upon activation, resulting in the reorganization of the actin network [44], which leads to the intracellular distribution of SAV1 and MST1/2 kinases. The remodeling alters the localization of these signaling molecules in the cell, leading to notable differences in their distribution across the cell membrane, cytoplasm, and nucleus [45,46]. MST1/2 and SAV1 are concentrated in specific regions following reorganization, increasing the likelihood of their interaction.

### 2.4. MST1/2-LATS1/2-MOB1 Axis in Hippo Signaling Pathway

MST1/2-SAV1 complex directly phosphorylates conserved serine or threonine residues on LATS1/2. The main phosphorylation site in LATS1 is Thr1079, and it is Thr1041 in LATS2. Phosphorylation at these sites triggers conformational changes in LATS1 and LATS2, relieving their self-inhibitory mechanisms and enhancing their ability to phosphorylate downstream substrates [47]. Subsequently, the phosphorylated LATS1/2 recruits Mps one binder 1 (MOB1), facilitating the interaction between MOB1 and LATS1/2.

The MOB protein family, which includes MOB1A and MOB1B in human cells, is the crucial component of the Hippo signaling pathway. These proteins specifically interact with the N-terminal regulatory region (NTR) of LATS1/2 kinases through a conserved domain. This interaction is finely tuned, with the Asp63 residue on MOB1 being essential for stable binding to LATS1/2; moreover, Lys104 and Lys105 are critical for the interaction between MOB1 and MST1/2. MST1/2 activation phosphorylates MOB1A/B at key sites, particularly T12 and T35, which enhances the affinity of MOB1A/B for LATS1/2, facilitating the formation of a more stable LATS1/2-MOB1A/B complex. This complex stabilizes the conformation of LATS1/2 and promotes their full activation, increasing their kinase activity and leading to the phosphorylation and functional regulation of downstream effectors such as YAP/TAZ [48]. Once LATS1/2 are fully activated, they phosphorylate YAP/TAZ, thereby inhibiting their original biological functions and redirecting cellular activities toward different pathways.

### 2.5. YAP/TAZ in the Hippo Signaling Pathway

YAP and TAZ are essential effectors in the Hippo signaling pathway, pivotal for regulating cell fate, tissue growth, and organ size. YAP/TAZ possess NLS recognized by nuclear import receptors. When YAP/TAZ are unphosphorylated by kinases such as LATS1 or LATS2, YAP/TAZ are able to enter the nucleus via the nuclear pore complex, which is vital for their functional activity [49]. When YAP/TAZ are transported to the nucleus, they interact with transcription factors from the TEA Domain (TEAD) family, forming a powerful transcriptional regulatory complex. TEAD family includes TEAD1 to TEAD4, which are conserved across a wide range of organisms, from yeast to humans, and belong to the TEA/ATTS transcription factor family. TEAD proteins are characterized by a distinctive TEA domain that enables them to recognize and bind to TEAD response elements (TREs) containing the TGTCTC sequence, which regulates gene expression and influences key biological processes such as the cell cycle, differentiation, and apoptosis [50]. The TEAD-binding domain (TBD) is the N-terminal TEAD-binding domain of YAP and TAZ, allowing them to recruit TEADs to specific gene promoters or enhancer regions [51]. This interaction stabilizes the structure of TEAD and significantly enhances its transcriptional activity [50], which leads to the upregulation of genes associated with cell proliferation, migration, and survival, including connective tissue growth factor (CTGF), cysteine-rich angiogenic inducer 61 (CYR61), and annexin A2 (ANX2) helping cells to respond to external signals, maintain tissue homeostasis, and is closely related to various pathological conditions, such as fibrosis and cancer [51].

Once the LATS1/2-MOB1A/B complex is formed, the activity of the LATS1/2 kinases is fully activated, enabling them to phosphorylate YAP/TAZ, which possess numerous phosphorylation sites, and different kinases can phosphorylate specific sites, leading to distinct functional outcomes [52,53]. Specifically, the LATS1/2 kinases directly phosphorylate YAP at serine 127 (Ser127) [54] and serine 381 (Ser381) [55], as well as TAZ at serine 89 (Ser89) [56] and serine 311 (Ser311) [57], which alters the conformation of YAP/TAZ. Existing literature has demonstrated that the formation of p-YAP (S381) and p-TAZ (S311) facilitates subsequent phosphorylation and is involved in the ubiquitination and degradation of YAP and TAZ [55,57]. In contrast, the phosphorylation of p-YAP (S127) and p-TAZ (S89) exposes binding motifs for 14-3-3 proteins, typically RSXpSXP (mode 1) or RXXXpSXP (mode 2), promoting their interaction with 14-3-3 proteins, which ultimately retains YAP/TAZ in the cytoplasm, preventing their translocation to the nucleus and inhibiting their function as transcriptional co-activators [54,56]. The 14-3-3 proteins contain two binding pockets that interact with phosphorylated serine residues and adjacent amino acids. Under these conditions, 14-3-3 proteins bind to phosphorylated YAP/TAZ, altering their structure and masking the NLS, which inhibits their ability to enter the nucleus and bind to TEADs, furthermore regulating excessive cell proliferation and promoting appropriate apoptosis [54,56]. Phosphorylated YAP/TAZ, particularly p-YAP (S381) and p-TAZ (S311) that remain in the cytoplasm, can no longer enter the nucleus to perform their functions. Cells employ specific pathways to maintain a certain level of YAP/TAZ in the cytoplasm. Current research indicates that these retained p-YAP/p-TAZ have two primary fates: (1) one major way is ubiquitin-mediated degradation. YAP/TAZ in the cytoplasm can be recognized and phosphorylated by casein kinase 1 delta/epsilon (CK1δ/ε) at additional sites near the already phosphorylated residues by LATS1/2 [for p-YAP (S381), including Ser384 and Ser387]. This series of consecutive phosphorylation events creates a unique sequence known as a phosphodegron [58]. SCFβ-TRCP is part of an E3 ubiquitin ligase complex that recognizes phosphorylated proteins. Once SCFβ-TRCP identifies and binds to the phosphorylated YAP/TAZ, it catalyzes their polyubiquitination, leading to ubiquitin-mediated degradation of these proteins. This process ensures dynamic equilibrium of YAP/TAZ levels within the cell [55,57,58]; (2) the other fate for YAP/TAZ involves their interaction with the AMOT protein family and the crumbs (Crb) complex, which results in their accumulation near the cell membrane. The AMOT protein family consists of proteins that contain multiple PDZ domains, while the Crb complex is crucial for cell polarity and the formation of tight junctions. Members of the AMOT family can directly interact with phosphorylated YAP/TAZ, anchoring them to the cell membrane through the Crb complex, thereby reducing their chances of entering the nucleus and inhibiting their co-activator function in transcription [59]. Additionally, this interaction helps maintain apicobasal polarity in the cell. The preservation of this polarity further promotes the interaction of YAP/TAZ with AMOT, enhancing membrane translocation of YAP/TAZ [60]. Therefore, this binding is crucial for maintaining cell polarity, facilitating the formation of tight junctions, and regulating the Hippo signaling pathway.

## 3. ECM and Hypoxia 

The TME is a complex entity surrounding tumors, comprising cellular components like fibronectin and various non-cellular elements such as ECM, oxygen levels, pH, cytokines, growth factors, and metabolic byproducts. It is widely recognized that the TME plays a crucial role in tumor progression, invasion, and treatment response [61], and changes in the TME composition can significantly alter cellular physiological behavior. The non-cellular components of the TME significantly influence the physiological behavior and invasiveness of tumor cells. Factors such as ECM, cytokines, pH, oxygen concentration, growth factors, and metabolic byproducts have been extensively studied for their impacts on tumor development and interactions between tumor cells. Notably, there exists a complex interplay between hypoxia and the ECM, which profoundly affects cellular behavior and physiological/pathological processes in tissues. Under hypoxic conditions, the expression of MMPs in tumor cells can be significantly upregulated, promoting ECM remodeling and enhancing invasiveness and metastatic potential. In addition to inducing structural changes in the ECM, hypoxia can also lead to compositional alterations, including increased levels of hyaluronic acid (HA) and excessive deposition of fibronectin and laminin [5,6,62]. These compositional changes increase the rigidity of the ECM and transmit signals to cells through integrin-mediated signaling pathways, activating downstream signals such as HIFs to regulate cell adhesion, proliferation, and migration [6,62]. Furthermore, abnormal ECM remodeling is generally believed to affect angiogenesis, exacerbating the local hypoxic environment and stabilizing the hypoxic microenvironment within the tumor over time.

### 3.1. ECM in Breast Cancer

The ECM is a complex network composed of various biomolecules, including collagen, elastin, glycosaminoglycans, proteoglycans, and fibronectin [63]. This three-dimensional environment provides structural support to cells and transmits signals to them via surface receptors, regulating diverse cellular behaviors and playing a critical role in physiological processes such as adhesion, proliferation, differentiation, migration, and apoptosis [63,64,65,66]. ECM remodeling has a decisive impact on the invasion, metastasis, and angiogenesis of tumor cells under pathological conditions. For instance, the overexpression of MMPs in pathological states can degrade key ECM components, thereby enhancing the invasive and metastatic capabilities of tumor cells [63,65,67]. Therefore, the ECM not only serves as a physical scaffold providing essential support to cells but also significantly influences the onset and progression of diseases through its dynamic remodeling and restructuring.

#### 3.1.1. ECM Stiffness in Pathological Process

**Figure 1:**
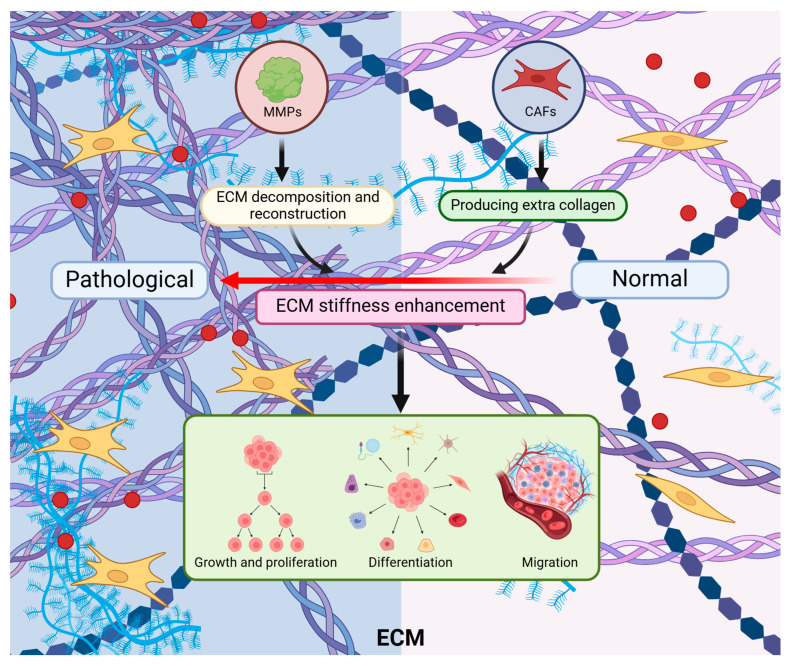
Normal and pathological state of the ECM. Cancer-Associated Fibroblasts (CAFs) overexpress collagen and remodel the extracellular matrix (ECM), resulting in increased stiffness of the ECM, which further promotes the transition of normal cells to the pathological state. Matrix metalloproteinases (MMPs) facilitate the degradation and remodeling of the ECM, which in turn affects stiffness. Additionally, the increased stiffness of the ECM affects various aspects, including tumor cell proliferation, differentiation, and migration. Created with BioRender.com.

The stiffness of the ECM is a crucial component of tissue mechanical properties and is closely linked to cellular behavior, tissue function, signaling pathways, and the onset and progression of diseases [68,69]. In normal cells, the stiffness of the ECM is typically maintained within an optimal range to support normal growth, differentiation, and migration [63]. The appropriate stiffness is achieved through the interactions and dynamic balance of ECM components [66]. However, the stiffness of the ECM can change significantly in pathological states. Particularly, ECM stiffness is often higher than that of normal tissues in tumor tissues, a phenomenon referred to as “stiffening” [65]. The deposition of collagen varies significantly under both physiological and pathological conditions. Normally, collagen, as a primary component of the ECM, is essential for maintaining tissue structure and function [70]. In the TME, the levels and activities of MMPs are often altered, facilitating the degradation and remodeling of the ECM, which in turn affects its stiffness. Additionally, various factors within the TME may activate cancer-associated fibroblasts (CAFs), leading to an increased synthesis and accumulation of collagen, particularly type I and type III. Furthermore, the cross-linking between collagen molecules is enhanced, stabilizing the ECM structure and increasing its stiffness (Figure 1).

#### 3.1.2. ECM Stiffness in Breast Cancer

**Figure 2:**
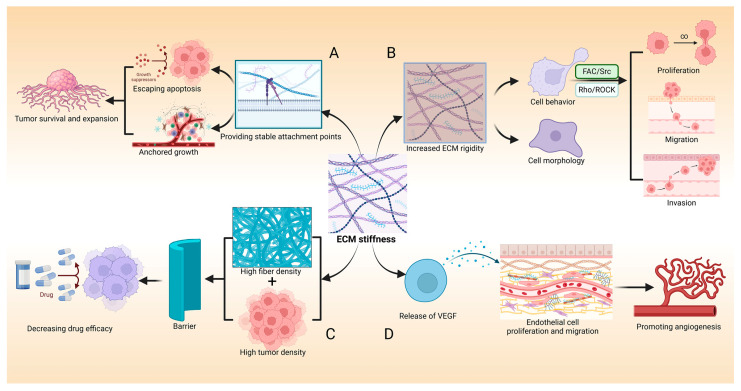
Effects of the ECM stiffness on breast cancer cells. (**A**) Increased stiffness of the ECM provides stable adhesion points for tumor cells, aiding their escape from apoptosis and supporting sustained growth, thereby promoting tumor survival and expansion. (**B**) The increased rigidity of the ECM influences tumor cells through both cellular behavior and morphology, ultimately facilitating tumor dissemination, migration, and invasion. (**C**) A highly condensed ECM is characterized by high fiber density and high tumor density, creating a dense barrier that impedes drug penetration into tumor tissue, resulting in reduced drug efficacy. (**D**) A dense ECM promotes the release of VEGF by cells, which accelerates the proliferation and migration of vascular endothelial cells, ultimately enhancing angiogenesis. Created with BioRender.com.

The interaction between the ECM and tumor cells plays a critical role in tumor initiation, progression, and survival and metastasis. The stiffening of the ECM not only facilitates the formation of the TME but also influences tumor cell proliferation, migration, and invasion through its biomechanical properties [3] (Figure 2A). ECM stiffening alters its biomechanical characteristics, leading to increasing rigidity, which can directly affect the morphology and behavior of tumor cells [66] (Figure 2B). For instance, stiffer ECM can activate integrins and other mechanosensitive receptors, triggering intracellular signaling pathways such as FAK/Src and Rho/ROCK, which are closely associated with cell proliferation, migration, and invasion [71]. Additionally, the stiffened ECM provides stable adhesion points, enhancing tumor cell attachment to the matrix, which helps tumor cells resist anoikis and increases their survival under unfavorable conditions. The capacity of enhanced adhesion also promotes anchorage-dependent growth, laying the foundation for the sustained expansion of the tumor [72].

The stiffened ECM significantly increases the density of tumor tissue and the complexity of the fibrous network, forming a physical barrier [3,73], which hinders the effective infiltration of immune cells, weakens their cytotoxic effects, and increases the internal pressure within the tumor. It further obstructs the uniform distribution of drugs within the tumor, potentially exposing some tumor cells to lower drug concentrations and reducing the therapeutic efficacy [74,75] (Figure 2C). On the other hand, changes in the ECM promote tumor angiogenesis, providing essential nutrients and oxygen to the tumor. It is primarily because the remodeling and stiffening of the ECM lead to the release of angiogenic factors, such as VEGF, that were previously bound in the ECM [3,76]. VEGF can promote new blood vessel formation, capable of activating vascular endothelial cells to stimulate their proliferation and migration. Besides, the ECM provides a supportive environment that allows vascular endothelial cells to migrate and proliferate in a directional manner along the ECM fibers. Specific components of the ECM, such as collagen and laminin, can serve as tracks for the migration of vascular endothelial cells, which provides the necessary physical support and directional cues [77,78] (Figure 2D). The occurrence of these events indicates that changes in ECM stiffness have profound effects on tumor initiation and progression.

#### 3.1.3. ECM and Hippo Signaling Pathway

When the stiffness of the ECM increases, it can be detected by mechanoreceptors on the cell surface [79], with integrins being the most prominent receptors [72,80]. Integrins are transmembrane proteins that link cells to the ECM and relay mechanical signals from the extracellular environment into the cell [72,79]. Integrins act as ligands for various ECM components, such as fibronectin and laminin [79,81]. Upon binding to these ECM components, a specialized structure known as focal adhesions forms at specific regions of the cell membrane, primarily at the contact sites between cells and the ECM [72]. Within these focal adhesions, integrins interact with cytoskeletal elements and activate a cascade of downstream signaling molecules [80], including FAK found in the cytoplasm [82]. FAK is a non-receptor tyrosine kinase that is vital in mechanotransduction [83]. When integrins bind to ECM ligands, FAK is recruited to focal adhesions, where the tyrosine residue Y397 undergoes phosphorylation [72,82]. This autophosphorylation event is the initial step of FAK activation, facilitating a conformational change from an inactive to an active state [84]. Moreover, phosphorylation at the Y397 site serves as a binding site for Src family kinases (SFKs), which can interact with FAK [83]. Once SFKs bind to the Y397 site, they further phosphorylate additional tyrosine residues on FAK, particularly Y576 and Y577 [85]. This cascade of phosphorylation events leads to the full activation of FAK, initiating phosphorylation of its downstream targets and consequently triggering various signaling pathways that regulate cellular processes such as proliferation, migration, survival, and differentiation [82,83]. Multiple studies have reported that FAK activation can influence the Hippo signaling pathway through various mechanisms [86,87]. These pathways include direct or indirect effects on YAP/TAZ by regulating the activity of Rho GTPase family members, the PI3K/Akt pathway, and other signaling molecules [88,89]. Current research indicates that activated FAK can directly phosphorylate specific sites on YAP/TAZ, altering their phosphorylation state and consequently modulating their binding affinity for 14-3-3 proteins. The interaction promotes the binding of YAP/TAZ to 14-3-3, preventing their translocation into the nucleus and ultimately regulating cellular processes such as proliferation, differentiation, and apoptosis [87,90]. The Rho GTPase family is a group of small GTP-binding proteins that are part of the larger Ras superfamily [91]. These proteins primarily function as molecular switches, regulating cytoskeletal remodeling to alter cell shape and motility [92]. The dynamic changes in the cytoskeleton, particularly in actin filaments, influence cell-cell contact and adhesion, thereby affecting cell polarity and morphology, as well as the formation of tight and gap junctions [93]. FAK plays a crucial role in the regulation of cytoskeletal remodeling by modulating the activity of Rho GTPase family members, including RhoA, Rac1, and Cdc42 [94]. Specifically, Rho GTPases are regulated by various upstream factors, including guanine nucleotide exchange factors (GEFs) and GTPase-activating proteins (GAPs) [92]. GEFs promote the conversion of Rho GTPases from a GDP-bound state to an active GTP-bound state, activating Rho GTPase family proteins. Conversely, GAPs facilitate GTP hydrolysis, returning Rho GTPases to their inactive state [92]. FAK can phosphorylate specific GEFs, such as p115-RhoGEF, regulating the activity of Rho GTPases and promoting RhoA activation, which subsequently leads to actin filament polymerization and cytoskeletal remodeling [94]. VAV2, a GEF, significantly influences cell migration and morphological changes by activating Rac1 and Cdc42. Besides, FAK can regulate Rho GTPase activity by phosphorylating specific GAPs, further affecting their function [94].

Cytoskeletal remodeling is vital not only in cell shape and motility but also in regulating the distribution and activity of intracellular signaling molecules, including those involved in the Hippo signaling pathway [95,96]. Specifically, cytoskeletal reorganization impacts the localization and function of key molecules in the Hippo pathway, YAP/TAZ. FAK activates RhoA, Rac1, and Cdc42 by phosphorylating specific GEFs, including p115-RhoGEF and VAV2, which subsequently induce actin filament polymerization and cytoskeletal remodeling, affecting cell morphology and adhesion. These changes in the cytoskeleton can promote the nuclear localization of YAP/TAZ, thereby activating downstream gene expression in the Hippo pathway. Moreover, cytoskeletal reorganization influences the activity of upstream molecules in the Hippo pathway, such as MST1/2 and Sav1 [97]. These upstream molecules are typically activated in a contact-dependent manner, and the dynamic changes in the cytoskeleton can enhance or inhibit the contact-dependent activation, subsequently influencing the activity of the Hippo signaling pathway [96,97].

**Figure 3:**
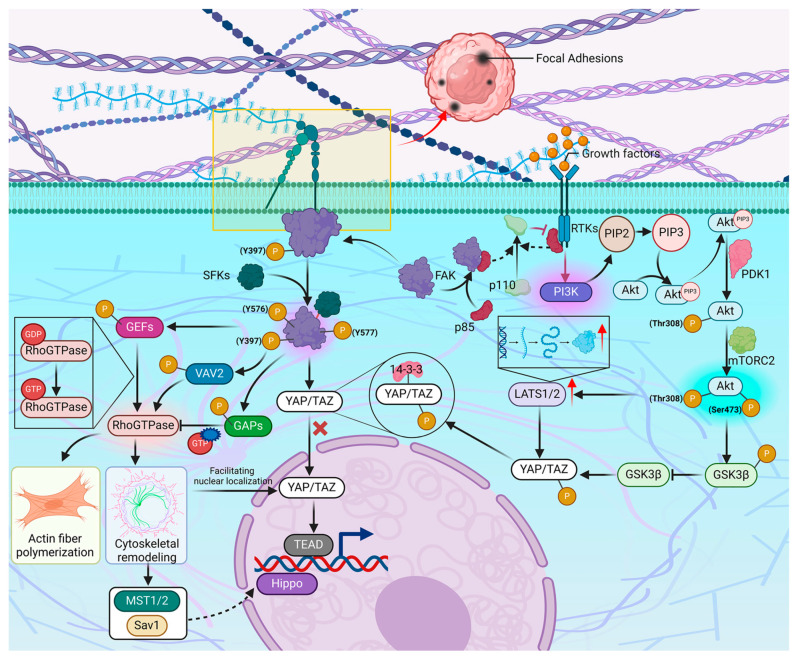
Intracellular signaling regulation of the ECM signal transmission. The extracellular matrix (ECM) conveys signals into cells via focal adhesions, initially phosphorylating FAK at the Y397 site. Assisted by Src family kinases (SFKs), FAK is further phosphorylated at the Y576 and Y577 sites, leading to its full activation. Activated FAK then phosphorylates guanine nucleotide exchange factors (GEFs), VAV2, and GTPase-activating proteins (GAPs), thereby activating Rho GTPases, which remodel the cytoskeleton and inhibit fiber polymerization, ultimately regulating the activity of the Hippo signaling pathway within the cell. Besides, ECM can activate the PI3K/Akt signaling pathway by releasing stored growth factors that bind to receptor tyrosine kinases (RTKs) on the cell membrane. Akt influence protein expression through the mTORC1 pathway, leading to an increased LATS1/2 expression, which ultimately inhibits the nuclear translocation of YAP/TAZ. Conversely, when Akt phosphorylates and inhibits GSK3β, the reduction of YAP/TAZ phosphorylation allows them to evade 14-3-3 protein binding, facilitating their entry into the nucleus to perform functions, which highlights the dynamic and complex nature of biological systems. Created with BioRender.com.

The PI3K/Akt signaling pathway is an intracellular signaling cascade that is critical in cellular responses to external signals, particularly in processes such as metabolism, proliferation, survival, growth, and angiogenesis [98], and it primarily consists of two key molecules: the phosphoinositide 3-kinase (PI3K) complex and Akt, whose activation relies on the collaborative action of multiple upstream signals. Receptor tyrosine kinases (RTKs), which are transmembrane proteins located on the cell membrane, play a pivotal role in the activation of the PI3K/Akt signaling pathway [99]. When ligands such as growth factors or hormones bind to RTKs on the cell surface, these receptors undergo dimerization and autophosphorylation, creating binding sites for other signaling molecules. At this point, the regulatory subunit p85 of PI3K can bind to the activated RTKs, which brings the catalytic subunit p110 close to the cell membrane, and once p110 is in proximity to the membrane, p85 releases its inhibitory effect on p110, leading to the activation of PI3K [99]. In addition, studies have shown that FAK can bind to the p85 subunit, which subsequently recruits the p110 subunit [88]. The activated PI3K complex catalyzes the conversion of phosphatidylinositol 4,5-bisphosphate (PIP2) to phosphatidylinositol 3,4,5-trisphosphate (PIP3) on the cell membrane. The generated PIP3 can bind to the pleckstrin homology (PH) domain of Akt, leading to the translocation of Akt from the cytoplasm to the cell membrane. Once localized to the membrane, Akt undergoes partial phosphorylation at the Thr308 site by 3-phosphoinositide-dependent protein kinase-1 (PDK1). However, Akt is not fully active until it is phosphorylated at the Ser473 site by mammalian target of rapamycin complex 2 (mTORC2), which completes its activation. Activated Akt exerts multiple effects on the Hippo signaling pathway and has an essential role in cell proliferation, survival, and apoptosis [98]. Research indicates that Akt promotes protein translation through the mTORC1 pathway, potentially increasing the expression of LATS1/2 and enhancing the phosphorylation of YAP/TAZ, leading to their retention in the cytoplasm and subsequent degradation. Glycogen synthase kinase 3 beta (GSK3β) is a multifunctional serine/threonine kinase critical for metabolism, development, differentiation, and signaling transduction. Under normal conditions, GSK3β phosphorylates YAP/TAZ, causing them to bind to 14-3-3 proteins, which prevents their nuclear translocation and results in their accumulation in the cytoplasm, ultimately leading to ubiquitin-mediated degradation. Akt can inhibit GSK3β activity by phosphorylating it, consequently reducing YAP and TAZ phosphorylation [100], promoting their entry into the nucleus to interact with TEAD family members, and enhancing their transcriptional activity (Figure 3).

#### 3.1.4. miRNAs Targeting Fibronectin in the ECM

**Figure 4:**
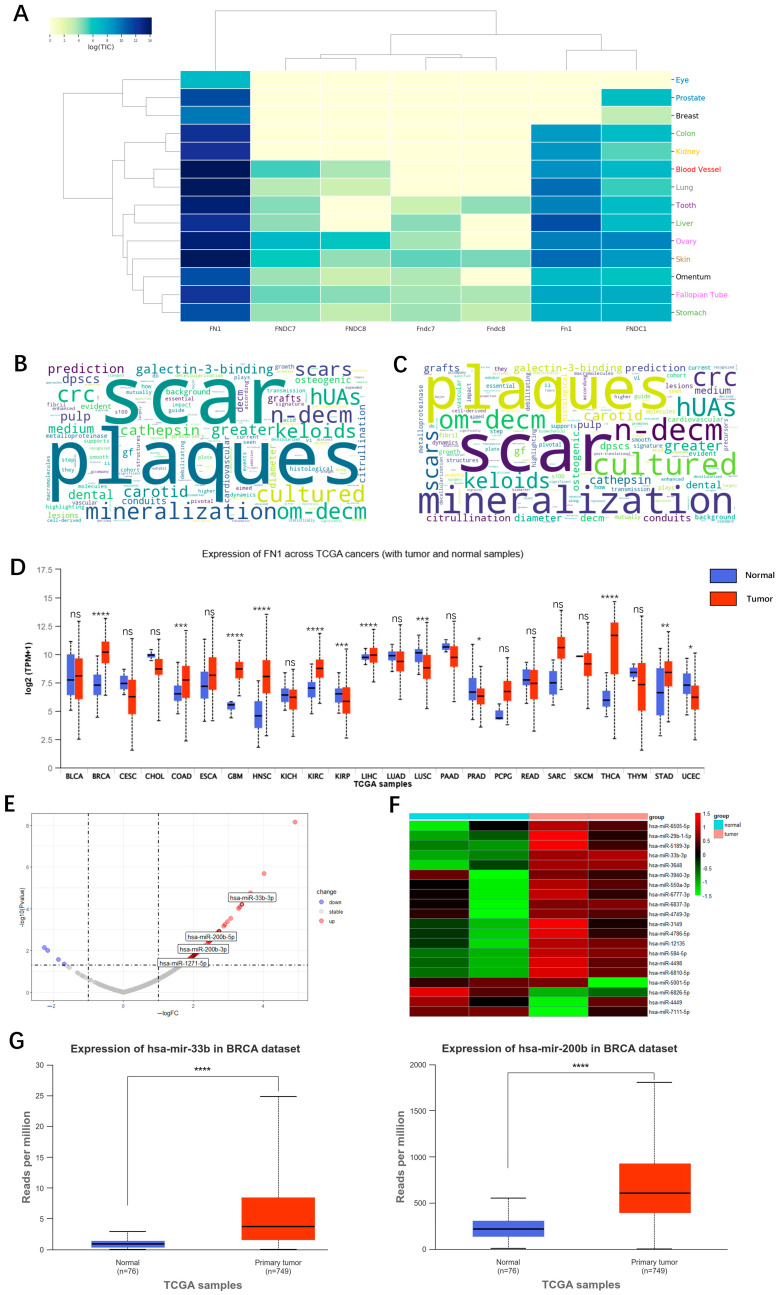
Identification of miRNAs targeting fibronectin in the ECM of breast cancer. (**A**) A heatmap illustrating fibronectin-related gene expression across various tissues was generated using the keyword “Fibronectin” in the MatrisomeDB database, revealing that FN1 exhibited the most significant differential expression in breast tissue. (**B**,**C**) Functional word cloud diagrams obtained by searching for “Fibronectin” and “FN1” in MatrisomeDB indicate that both fibronectin and FN1 are associated with the dECM. (**D**) Pan-cancer analysis conducted via the UALCAN database (https://ualcan.path.uab.edu/cgi-bin/Pan-cancer.pl?genenam=FN1, accessed on 2 August 2024) demonstrated significant expression differences for FN1, particularly notable in breast cancer. BLCA, Bladder urothelial carcinoma (n = 19 normal samples, n = 408 primary tumors); BRCA, Breast invasive carcinoma (n = 114 normal samples, n = 1097 primary tumors); CESC, Cervical squamous cell carcinoma (n = 3 normal samples, n = 305 primary tumors); CHOL, Cholangiocarcinoma (n = 9 normal samples, n = 36 primary tumors); COAD, Colon adenocarcinoma (n = 41 normal samples, n = 286 primary tumors); ESCA, Esophageal carcinoma (n = 11 normal samples, n = 184 primary tumors); GBM, Glioblastoma multiform (n = 5 normal samples, n = 156 primary tumors); HNSC, Head and neck squamous cell carcinoma (n = 44 normal samples, n = 520 primary tumors); KICH, Kidney chromophobe (n = 25 normal samples, n = 67 primary tumors); KIRC, Kidney renal clear cell carcinoma (n = 72 normal samples, n = 533 primary tumors); KIRP, Kidney renal papillary cell carcinoma (n = 32 normal samples, n = 290 primary tumors); LIHC, Liver hepatocellular carcinoma (n = 50 normal samples, n = 371 primary tumors); LUAD, Lung adenocarcinoma (n = 59 normal samples, n = 515 primary tumors); LUSC, Lung squamous cell carcinoma (n = 52 normal samples, n = 503 primary tumors); PAAD, Pancreatic adenocarcinoma (n = 4 normal samples, n = 178 primary tumors); PRAD, Prostate adenocarcinoma (n = 52 normal samples, n = 497 primary tumors); PCPG, Pheochromocytoma and paraganglioma (n = 3 normal samples, n = 179 primary tumors); READ, Rectum adenocarcinoma (n = 10 normal samples, n = 166 primary tumors); SARC, Sarcoma (n = 2 normal samples, n = 260 primary tumors); SKCM, Skin cutaneous melanoma (n = 1 normal samples, n = 472 primary tumors); THCA, Thyroid carcinoma (n = 59 normal samples, n = 505 primary tumors); THYM, Thymoma (n = 2 normal samples, n = 120 primary tumors); STAD, Stomach adenocarcinoma (n = 34 normal samples, n = 415 primary tumors); UCEC, Uterine corpus endometrial carcinoma (n = 35 normal samples, n = 546 primary tumors). * *p* < 0.05; ** *p* < 0.01; *** *p* < 0.001; **** *p* < 0.0001; ns = non-significant. (**E**) Differentially expressed genes were identified with |log_2_ FC| ≥ 1 and *p* < 0.05 as thresholds. Differential analysis was performed using the limma package, and the results indicated that the selected miRNAs were all upregulated. (**F**) A heatmap was generated for the top ten upregulated miRNAs in the GSE224147 dataset, with hsa-miR-33b-3p ranking in the top ten on expression levels. (**G**) Analysis using the UALCAN database revealed that both hsa-miR-33b-3p and hsa-miR-200b exhibited upregulated expression in breast cancer cells [101,102,104,109,110].

Fibronectin is a large fibrous glycoprotein widely found in the animal kingdom, playing crucial roles in cell migration, adhesion, proliferation, hemostasis, and tissue repair. As a key component of the ECM, changes in fibronectin conformation and distribution during ECM stiffening significantly impact tumor development. In breast cancer, ECM stiffening is closely associated with alterations in fibronectin. Signals from the stiffened ECM transmitted by fibronectin can promote the invasion and metastasis of tumor cells. Therefore, identifying and targeting key molecules that modulate fibronectin function is essential for developing novel therapeutic strategies for breast cancer treatment. It is valuable to discover a molecule serving as a new therapeutic target that can interfere with fibronectin activity, in this way inhibiting breast cancer progression and providing innovative solutions for clinical treatment. Thus, we conducted a preliminary screening of fibronectin-related genes using the MatrisomeDB database and identified several relevant genes [101], among which FN1 showed the most significant differential expression in breast tissue (Figure 4A). FN1 encodes fibronectin, which is material for scar formation and in the abnormal deposits or lesions (plaques) that form on or within tissues (Figure 4B). In addition, FN1 is strongly correlated with the decellularized extracellular matrix (dECM) (Figure 4C). Results from UALCAN indicate that FN1 expression levels are higher in various types of tumor tissues compared to adjacent tissues (Figure 4D) [102]. Although there is currently no direct literature reporting it, it is highly likely that FN1 influences breast cancer cell activity through its effects on the ECM.

miRNAs (microRNAs) are a class of endogenous small non-coding RNA molecules, typically consisting of 19-24 nucleotides. They are widely present in eukaryotes and play a crucial role in regulating gene expression, significantly impacting biological processes such as cell differentiation, proliferation, and apoptosis [103]. Several studies have reported that miRNAs can influence FN1 expression, including hsa-miR-33b-3p [104], the hsa-miR-200 family (comprising has-200b [105] and has-miR-200c [106,107]), as well as hsa-miR-1271-5p [108]. These miRNAs impact the biological behavior and therapeutic outcomes of breast cancer by altering cell invasion capacity, modifying sensitivity to targeted chemotherapy drugs, and activating the PI3K/Akt signaling pathway.

To further investigate the role of these miRNAs in breast cancer, we analyzed the GSE224147 dataset [109], which contains miRNA expression data from immortalized endothelial cell lines derived from breast cancer patients under hypoxic conditions. The analysis revealed that the four miRNAs of interest mentioned above were all upregulated in this dataset (Figure 4E), with hsa-miR-33b-3p showing particularly notable differential expression, capturing our attention (Figure 4F). Further analysis using the UALCAN database indicated that hsa-miR-33b-3p is significantly upregulated in BRCA (Figure 4G). Furthermore, we utilized the RNA22 database [110] to predict the target genes of hsa-miR-33b-3p, which identified FN1 as a potential target, suggesting that hsa-miR-33b-3p has the possibility to regulate FN1 function.

In sum, FN1 is a crucial protein in the ECM, playing an essential regulatory role in cell adhesion, migration, and proliferation. It serves as an important bridge between the ECM and intracellular signaling. miRNAs are generally believed to regulate gene expression by binding to the 3′ untranslated region (3′ UTR) of mRNAs, which typically leads to mRNA degradation or reduced translation efficiency. Given that hsa-miR-33b-3p directly targets FN1 mRNA, it could theoretically bind to the 3′ UTR region of FN1 mRNA, inhibiting its translation into protein and thereby reducing FN1 levels within the cell, which would ultimately modulate ECM signaling to the cell. Consequently, hsa-miR-33b-3p has significant potential as a novel therapeutic target for breast cancer.

### 3.2. Hypoxia in Breast Cancer

Oxygen concentration is another important non-cellular factor that exhibits heterogeneous distribution in the TME. The hypoxic condition arises from the imbalance between the rapid growth of tumor tissue and the supply from the vascular system [111]. In particular, the incomplete and uneven distribution of newly formed blood vessels leads to hypoxic regions in solid tumors [112,113,114], activating various signaling pathways, primarily by stimulating the HIFs family, which promotes the expression of angiogenic factors, thereby stimulating the formation of new blood vessels [112,113,115]. Tumor cells undergo metabolic reprogramming in hypoxia, preferentially relying on glycolysis for energy production, resulting in local lactic acid accumulation, creating an acidic microenvironment that impacts cellular functions and therapeutic responses [112,114,115]. ECM undergoes significant changes in hypoxia, with excessive accumulation of fibronectin and laminin, leading to increased ECM stiffness, which can influence cell adhesion and migration capabilities via integrin-mediated pathways [4,116,117]. Furthermore, it is evident that oxygen concentration can regulate YAP/TAZ activity by affecting HIF-1α [118,119].

#### 3.2.1. HIFs and Hippo Signaling Pathway

**Figure 5:**
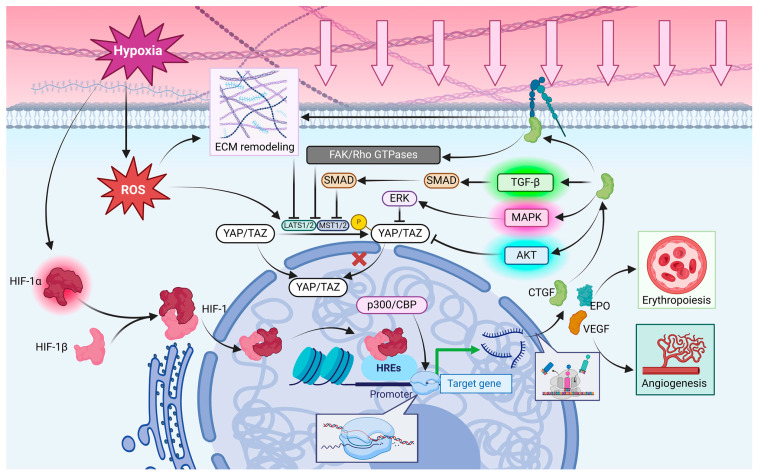
Hypoxia regulates intracellular signaling through multiple mechanisms. Under hypoxic conditions, hypoxia-inducible factor 1-alpha (HIF-1α) stabilizes in the cytoplasm, dimerizing with HIF-1β to form HIF-1, which translocates to the nucleus, binding to hypoxia response elements (HREs) to promote the transcription of downstream genes, such as CTGF, EPO, and VEGF that regulate the process of tumor cells. CTGF can interact with ROS generated in the hypoxic environment via TGF-β pathway, MAPK pathway, and AKT signaling, ultimately affecting the upstream molecules of the Hippo signaling pathway and modulating YAP/TAZ function. Created with BioRender.com.

HIF-1α is the alpha subunit of hypoxia-inducible factor-1 (HIF-1), a heterodimer composed of HIF-1α and HIF-1β [119,120]. The activity and expression of HIF-1α are significantly dependent on the oxygen concentration in the environment. Under normoxic conditions, HIF-1α is hydroxylated by prolyl hydroxylases (PHDs), which require molecular oxygen as a substrate and target specific proline residues on HIF-1α. The hydroxylation allows VHL protein to recognize and tag HIF-1α, leading to its degradation via the ubiquitin-proteasome pathway, thus regulating HIF-1α levels in the cell. In hypoxic conditions, the reduced oxygen concentration decreases PHD activity, resulting in diminished hydroxylation of HIF-1α. HIF-1α cannot be recognized by VHL without hydroxylation, avoiding ubiquitination and degradation. Consequently, HIF-1α stabilizes and accumulates within the cell under hypoxic conditions [118,119,120]. Stable HIF-1α forms a heterodimer with the HIF-1β subunit in the cytoplasm, known as HIF-1 translocated to the nucleus [117,121], recognizing and binding to hypoxia response elements (HREs) located in the promoter regions of specific genes in the nucleus [122]. HIF-1 recruits transcriptional co-activators and other auxiliary proteins, such as p300/CBP, facilitating the assembly of the transcriptional complex and activating the transcription of downstream genes [119,121], which are primarily involved in adapting to hypoxic conditions, promoting processes such as angiogenesis, erythropoiesis, and metabolic reprogramming [121]. For example, HIF-1 can enhance the expression of VEGF and erythropoietin (EPO), promoting new blood vessel formation and red blood cell production and, as a result, improving oxygen supply to tissues [117]. Under hypoxic conditions, HIF-1α also plays a key role in recognizing and activating the CTGF gene, influencing the Hippo signaling pathway in cells (Figure 5).

The HIF family includes HIF-2α and HIF-3α in addition to HIF-1α. These proteins function as transcription factors, playing central roles in how cells sense and adapt to changes in oxygen levels. While research on HIF-1α is extensive, studies on the interactions between HIF-2α and HIF-3α with the Hippo signaling pathway are still relatively limited, and current reports on their effects on the Hippo pathway lack depth.

#### 3.2.2. Hypoxia and Hippo Signaling Pathway

Under hypoxic conditions, HIF-1α acts as a key transcription factor by recognizing and binding to specific DNA sequences in the promoter regions of target genes, known as HREs, to promote their transcription. This process enables cells to survive and adapt to low-oxygen environments [123]. CTGF is a multifunctional protein that plays significant roles in various physiological and pathological processes. The promoter region of the CTGF gene contains HREs, indicating that it can be directly recognized and activated by HIF-1α [124]. In hypoxia, HIF-1α binds to the HREs in the CTGF promoter, facilitating the synthesis of CTGF mRNA and its subsequent protein expression, which is crucial for cellular adaptation to low oxygen [125]. The upregulation of CTGF not only aids in cellular adaptation to hypoxic conditions but is also involved in fibrosis and cancer progression. CTGF can directly promote the remodeling of the ECM, which generally leads to increased ECM rigidity, which weakens the phosphorylation of YAP/TAZ by upstream kinases in the Hippo signaling pathway, such as MST1/2 and LATS1/2. Consequently, the retention of YAP/TAZ in the cytoplasm decreases, allowing for increased nuclear localization and activity of YAP/TAZ [124]. CTGF has been reported to indirectly influence the activity of YAP/TAZ by activating specific signaling pathways [124]. Notably, CTGF can activate the TGF-β signaling pathway, which plays a crucial role in cell proliferation, differentiation, and apoptosis. The TGF-β pathway transmits signals through the SMAD protein family, inhibiting the activity of upstream kinases in the Hippo pathway, such as MST1/2 and LATS1/2, reducing YAP/TAZ phosphorylation. Dephosphorylated YAP/TAZ is more likely to enter the nucleus and bind to TEAD transcription factors, activating the expression of downstream genes [126]. In addition, CTGF can bind to integrin receptors on the cell surface, activating integrin-mediated signaling pathways. Integrins transmit mechanical signals through interactions with the ECM, which can influence cell morphology and function. Key molecules in the integrin signaling pathway include FAK and Rho GTPases (RhoA) that can further impact cytoskeletal remodeling, further affecting the activity of YAP/TAZ. For instance, the activation of RhoA can inhibit kinases in the Hippo pathway, leading to decreased phosphorylation of YAP/TAZ [127]. CTGF can also activate the PI3K/Akt signaling pathway. Akt, also known as Protein Kinase B (PKB), is a key mitogen-activated protein kinase involved in cell survival, proliferation, and metabolism [128]. The activation of Akt can inhibit kinases in the Hippo pathway, such as MST1/2 and LATS1/2, reducing the phosphorylation of YAP/TAZ and facilitating their entry into the nucleus. Lastly, CTGF can activate the MAPK/ERK signaling pathway. ERK, a member of the MAPK family, regulates cell proliferation, differentiation, and survival [128]. The activation of ERK can also decrease the phosphorylation of YAP/TAZ by inhibiting Hippo pathway kinases, influencing their nuclear localization and transcriptional activity [126] (Figure 5).

Hypoxic conditions in the TME induce metabolic reprogramming in tumor cells, shifting their reliance from aerobic oxidation to a greater dependence on anaerobic glycolysis, potentially accompanied by adaptive changes in oxidative phosphorylation leading to increased production of reactive oxygen species (ROS) [129]. High concentrations of ROS are typically harmful to cells; however, tumor cells activate antioxidant defense mechanisms to counteract the toxic effects of ROS [129,130]. High ROS levels can affect the activity of the Hippo signaling pathway by altering the properties of the ECM or interacting with other cellular factors by promoting ECM remodeling and exacerbating fibrosis [131,132]. Elevated levels of ROS can directly or indirectly influence the phosphorylation status of YAP/TAZ, thereby altering their activity. It is indicated that ROS can promote the phosphorylation of YAP/TAZ, leading to their retention and degradation in the cytoplasm, which reduces their opportunity to enter the nucleus and activate downstream gene expression. Additionally, ROS may affect the activity of upstream proteins in the Hippo signaling pathway, such as LATS1/2 and MOB1A/B. One key mechanism by which ROS influences upstream proteins is through the oxidation of thiol groups (-SH), typically located on cysteine residues, affecting their function. For example, ROS can oxidize cysteine residues on MOB1, exposing interaction sites that promote its binding with the CREB-binding protein (CBP). Besides, other pathways are involved in the activation or inhibition of molecules, such as p38 MAPK or PI3K/Akt, which in turn affect the activity of LATS1/2 and MOB1A/B [131] (Figure 5).

## 4. Crosstalk of ECM and Hypoxia in Breast Cancer

**Figure 6:**
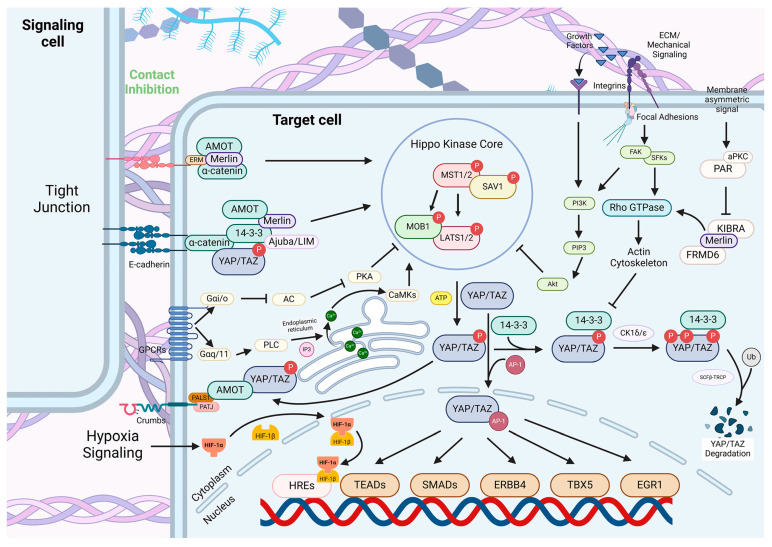
Crosstalk of ECM and hypoxia in the Hippo signaling pathway. The Hippo pathway can be influenced by various signals, including tight junctions, G protein-coupled receptors (GPCRs), the extracellular matrix (ECM), and hypoxia. Tight junctions modulate the activity of the Hippo core kinases by interacting with α-catenin and E-cadherin, furthermore impacting YAP/TAZ function. GPCRs can activate different G proteins, such as Gαi/o and Gαq/11, having distinct effects on the Hippo core kinases. The ECM transmits signals by altering the stiffness, releasing growth factors, and changing cell membrane polarity, which affects specific nodes in the Hippo pathway and alters the overall direction. Under normal conditions, YAP/TAZ are predominantly located in the cytoplasm, translocate to the nucleus via nuclear localization sequences, form complexes with AP-1, and bind to transcription factors such as TEADs to regulate downstream genes. The Hippo signaling pathway is activated when multiple upstream signals combined with the Hippo core kinases, leading to the phosphorylation of YAP/TAZ, which facilitates their binding to 14-3-3 proteins, preventing nuclear translocation and resulting in their ubiquitination and degradation or recruitment to the cell membrane. Under hypoxia, HIF pathway stabilizes HIF-1α, enabling it to dimerize with HIF-1β, facilitating HIF-1 entry into the nucleus to bind with HREs and regulate downstream gene expression. Created with BioRender.com.

ECM and hypoxia significantly influence the initiation and progression of breast cancer cells through complex interactions that markedly affect intracellular signaling, particularly the Hippo signaling pathway. Dysregulation of the Hippo pathway in breast cancer is closely associated with tumor progression. The combined effects of ECM and hypoxia impact the Hippo signaling pathway, thus significantly influencing the proliferation and invasive behavior (Figure 6).

The physical and chemical properties of the ECM are crucial in influencing the mechanosensing and signaling pathways of breast cancer cells [16]. Increased ECM stiffness under hypoxic conditions can activate downstream effectors of the Hippo signaling pathway, such as YAP/TAZ, promoting proliferation and invasion [5]. CAFs modify the ECM stiffness and structure by synthesizing and depositing ECM components, facilitating tumor cell invasion and metastasis. CAFs contribute to hypoxic conditions by consuming oxygen and secreting pro-angiogenic factors, further enhancing ECM deposition and stiffness [133]. Hypoxia activates the expression of genes related to angiogenesis, metabolic reprogramming, and cell survival through the stabilization of HIFs, collectively promoting malignant behaviors in breast cancer [2,134]. HIF-1α not only enhances the expression of angiogenic factors but also influences breast cancer progression by regulating the composition and physical properties of the ECM. HIF-1α promotes collagen crosslinking, increasing ECM stiffness and providing a more favorable mechanical environment for invasion and metastasis in breast cancer [6,16].

The interaction between the ECM and hypoxia has multifaceted effects on breast cancer cells [2]. Hypoxia-induced ECM remodeling increases ECM stiffness and alters its composition, creating a more favorable environment for proliferation and invasion [4]. Increased ECM stiffness enhances integrin signaling, activating FAK/Src kinases, which subsequently influence the activity of upstream kinases in the Hippo signaling pathway, such as MST1/2 and LATS1/2. Integrin signaling plays a crucial role in this process, as it resides at the interface between the ECM and cytoskeleton, guiding the transmission of signals from the ECM to breast cancer cells and participating in the regulation of the ECM stiffness and degradation [135,136].

Additionally, ECM remodeling provides breast cancer cells with enhanced survival and proliferation signals under hypoxic conditions, influencing the Hippo signaling activity by enhancing integrin signaling and activating related kinases [4]. The alterations in signaling can lead to the nuclear accumulation of YAP/TAZ, hence regulating the expression of a range of genes associated with growth and invasion in breast cancer [5,136].

### 4.1. ECM and Hypoxia Driving Tumor Metastasis

The ECM is a key component of the TME, and its physical and chemical properties significantly influence the mechanosensing and signaling of tumor cells. Increased ECM stiffness activates downstream effectors of the Hippo signaling pathway, such as YAP/TAZ, promoting tumor cell proliferation and invasion [5,137]. CAFs play a crucial role in regulating ECM behavior [138], as vital cellular components of the ECM, CAFs remodel the ECM by enhancing the expression of fibronectin and collagen, thereby altering the TME. In addition to physical remodeling, CAFs secrete various bioactive molecules, such as MMPs [137], to directly degrade and reshape the ECM, also releasing growth factors like VEGF and TGF-β that provide signals promoting tumor cell proliferation and invasion [138].

Moreover, CAFs exacerbate the hypoxic environment by consuming local oxygen and secreting angiogenic factors [137]. Hypoxia can activate transcription factors such as HIF-1α, which not only promotes the expression of genes related to angiogenesis and cell survival but also further enhances ECM stiffness by regulating its composition and physical properties, providing a more favorable mechanical environment for tumor cells. The activation of HIF-1α increases the expression of angiogenic factors and influences ECM degradation and remodeling, releasing growth factors that promote tumor angiogenesis and cell survival. This interaction forms a positive feedback loop, where CAFs and hypoxia collaboratively drive tumor invasion and metastasis [119,139,140]. It is evident that the interplay between ECM and these two factors constitutes a complex and critical network within the TME, collectively facilitating malignant progression and significantly impacting signaling regulation in tumor cells [5,137]. The interaction between ECM and hypoxic conditions significantly affects the Hippo signaling pathway, with key effectors YAP and TAZ being jointly regulated by ECM properties and hypoxic conditions, leading to profound effects on tumor cell behavior [90,141,142].

Under hypoxic conditions, the physical properties of the ECM change, characterized by increased matrix stiffness. This alteration may influence the activity of upstream kinases MST1/2 and LATS1/2 in the Hippo signaling pathway by enhancing integrin signaling and activating FAK/Src kinases. Integrin signaling plays a critical role at the interface between the ECM and the cytoskeleton, guiding signal transduction and participating in the regulation of ECM stiffness and degradation, thus providing a more favorable environment for tumor cell proliferation and invasion [90,141]. Additionally, the chemical composition of the ECM undergoes significant changes under hypoxia, including increased activity of MMPs, which leads to the degradation and remodeling of ECM components. This process releases growth factors such as VEGF and fibroblast growth factors (FGFs) binding to receptors on the cell surface, activating integrin signaling pathways and enhancing the adhesion and migration capabilities of tumor cells, which further influence the activity of the Hippo pathway, resulting in the accumulation of YAP/TAZ in the nucleus and the regulation of gene expression associated with tumor growth and invasion [141,143]. Furthermore, metabolic reprogramming under hypoxic conditions activates AMPK, which promotes nuclear accumulation of YAP/TAZ by inhibiting the mTORC1 signaling pathway. This interaction enhances the binding of YAP/TAZ to TEADs, activating the expression of genes that promote tumor growth, invasion, and metabolic adaptation, increasing the tumor cells’ resilience to a hypoxic environment. Specific transcription factors, such as CEBPD, enhance the invasiveness of tumor cells under hypoxia through ECM-integrin-mediated EGFR/PI3K signaling pathways. These transcription factors are important in the regulation of the Hippo signaling pathway by ECM and hypoxia, highlighting their critical roles in tumor cell adaptability and invasiveness [144].

### 4.2. ECM and Hypoxia Driving Tumor Metabolism Reprogramming

ECM influences cellular metabolism through various mechanisms, including but not limited to the regulation of growth factor and cytokine distribution, alteration of mechanical signaling pathways, modulation of cell adhesion and migration capabilities, and the regulation of intracellular metabolic pathways activity [145,146,147]. ECM provides the physical support necessary for cell survival and proliferation and directly affects the metabolic state of cells through its compositional components and structural characteristics, ultimately influencing cellular functions and fate [148,149].

ECM can bind and store various growth factors and hormones, influencing the metabolic state of cells by regulating the release of these factors. Specific components in the ECM, such as heparan sulfate proteoglycans (HSPGs) and laminin, efficiently bind to certain growth factors and hormones, serving as a storage function [146]. These components exhibit high hydrophilicity and negative charge, allowing for effective interaction with positively charged growth factors [150]. These growth factors and hormones primarily include FGF, epidermal growth factor (EGF), and insulin-like growth factor (IGF), which bind to the ECM in an inactive form, forming stable complexes. This binding provides a secure storage environment for growth factors and hormones, protecting them from degradation and ensuring their availability when needed. Besides, these factors can be released in response to cellular demands. For instance, ECM remodeling can trigger the release of growth factors, facilitating wound healing during tissue repair processes. The distribution of these components within the ECM is uneven, resulting in a heterogeneous binding pattern that helps confine growth factors and hormones to specific cellular microenvironments. The localization ensures that these factors exert their effects only in designated areas, allowing for precise regulatory outcomes. Moreover, the growth factors and hormones bound to the ECM are in an inactive state; they do not immediately elicit cellular responses, which allows cells to activate these factors at the appropriate time through specific signals (such as ECM remodeling or enzymatic action), thereby finely tuning cellular behavior [146]. Once growth factors and hormones bind to the ECM, they can interact with cell surface receptors, such as integrins, to synergistically amplify signal transduction effects, further influencing cellular metabolism and function [151]. Changes in the ECM or the action of specific enzymes, such as proteases, can lead to the release of these bound factors, activating downstream signaling pathways that affect cellular proliferation, differentiation, and metabolic states [152]. During the process of migration, cancer cells interact with the ECM, generating mechanical signals that induce deformation of the ECM. As the deformation occurs, the growth factors and hormones tightly bound within the ECM undergo conformational changes, facilitating their release from the ECM protein complex [5]. MMPs can specifically cleave proteins such as collagen and laminin, leading to structural changes in the ECM. During ECM remodeling, MMPs and other proteases, such as cathepsin B and cathepsin L, can be activated under specific conditions to cleave growth factors and hormones bound to the ECM. This process facilitates their release from the ECM, allowing them to bind to cell surface receptors and activate downstream signaling pathways, thereby influencing biological processes such as cell proliferation, migration, differentiation, and apoptosis [146,152].

In the TME, the ECM forms a three-dimensional network characterized by a porous structure. The three-dimensional environment plays a crucial role in maintaining tissue mechanical properties and enhancing cell-to-cell interaction. The pore sizes range from nanoscale to microscale, each serving distinct functions and exhibiting different shapes and permeabilities. These characteristics significantly influence cell–ECM interactions, including signaling pathways between them, affecting cell behavior. In normal tissues, ECM pores provide an environment for the uniform distribution of nutrients while facilitating the removal of metabolic byproducts like lactate, supporting normal cell growth and preventing toxic accumulation [148,153]. In the TME, ECM remodeling results in irregular pore structures, which hinder the effective transport of oxygen and nutrients, as well as the removal of metabolic waste. It leads to the formation of hypoxic regions and the accumulation of metabolic byproducts, adversely affecting cell function and the tissue microenvironment [5,148,154].

Specific components in the ECM, such as collagen and fibronectin, provide cells with distinct adhesion sites [155,156]. The adhesion state of cells to the ECM influences their morphology, which in turn affects their metabolic demands [157]. When cells are stably adhered to the ECM, they typically exist in a more quiescent state [155,157]. In this context, cells require more ATP to maintain structural stability and perform complex functions, leading them to rely on oxidative phosphorylation, an efficient energy production pathway, to meet their high energy needs [145,157]. In contrast, when cells migrate from one location to another, their adhesion to the ECM decreases, a state referred to as the migratory state [153]. In this scenario, cells require rapid energy supply to support processes such as cytoskeletal remodeling and cell movement. Consequently, cells tend to rely more on glycolysis to quickly generate the energy needed for these activities [157]. Similarly, the stiffening of the ECM makes it difficult for cells to form stable adhesion points, which impacts cytoskeletal rearrangement and leads to increased energy expenditure during the process of migration. To facilitate movement, cells adjust their metabolic pathways by enhancing glycolytic activity and reducing the reliance on oxidative phosphorylation, thus meeting the immediate energy demands required to overcome the resistance posed by the stiffer ECM [3].

When alterations of hypoxia and ECM coexist, tumor cells undergo more complex metabolic reprogramming. Under hypoxic conditions, tumor cells preferentially rely on glycolysis, and changes in ECM can further amplify this trend. For instance, the stiffening of the ECM enhances the Warburg effect through ROS/HIF-1α signaling, thereby driving tumor growth [158]. This mechanism involves chloride intracellular channel protein 1 (CLIC1), which enhances the stability of HIF-1α in tumor cells by inhibiting ROS hydroxylation, thereby increasing the glycolytic rate in tumor cells and providing more energy and biosynthetic precursors to support rapid proliferation and growth [159]. Hypoxia and changes in ECM stiffness can also regulate tumor cell metabolism by affecting autophagy, which is a degradation and recycling mechanism widely present in cells. When cells face adverse conditions such as nutrient deprivation, oxidative stress, infection, or aging, they initiate autophagy, forming autophagosomes to degrade damaged or aged components to obtain essential biomolecules and energy. These recovered molecules and energy can then be used to synthesize new proteins, organelles, and other necessary biomolecules, supporting cellular repair, reconstruction, and energy supply. In tumor cells, autophagy not only serves these functions but also is related to the adaptation to hypoxic conditions and changes in the ECM. When tumor cells detach from the ECM, they lose signals from it and initiate a programmed apoptotic pathway known as anoikis [160]. The integrin-GSK3β-FTO-mTOR axis is inhibited during ECM detachment, which enhances autophagy in tumor cells, helping them resist adverse environments and increasing survival rates [161].

Recent studies have increasingly focused on the individual and combined effects of ECM and hypoxia on tumor development. Although existing research indicates that ECM remodeling and hypoxic microenvironments are critical in enhancing tumor invasiveness, drug resistance, and immune evasion, there remain limits with many unresolved problems. Future research should prioritize investigating the roles of ECM and hypoxic conditions in tumor progression, as well as their joint effects on tumor physiological behaviors and drug resistance. It is necessary to develop new detection methods to accurately assess hypoxic status in the TME and further explore the key molecular mechanisms underlying ECM remodeling and CAF functions. Targeting critical factors involved in ECM remodeling or developing drugs to improve hypoxic conditions could offer new avenues for cancer treatment. Targeting MMPs, cathepsins, integrins, and HIF-1α have been extensively studied in breast cancer treatment, and they show promising potential for clinical application potential. 

## 5. Conclusions

The review discusses the key role of the Hippo signaling pathway in breast cancer, focusing on how the ECM and hypoxic microenvironment promote tumor cell proliferation, invasion, and metastasis by regulating relevant effector molecules, ultimately reducing tumor sensitivity to treatment. It reveals that miR-33b-3p may influence breast cancer progression by regulating the important ECM component FN1, providing a new direction for breast cancer therapy. However, although hsa-miR-33b-3p has been identified as a potential target of FN1, the analysis in the review is still in the early stages. Further in-depth exploration and research are needed to better understand its detailed effects, including further analysis using existing data and functional studies to confirm its impact.

Additionally, this review primarily focuses on the impact of the ECM and hypoxic microenvironment on the molecular mechanisms in breast cancer cells but does not delve into the clinical applications of these mechanisms or the roles of other components in the TME. As the TME is highly complex, other factors regulating the Hippo pathway require further investigation in addition to ECM and hypoxia. MMPs, other ECM-associated proteases, integrins, and HIF-1α are promising therapeutic targets for breast cancer. The structure and function of the ECM can be altered by modulating the activity of MMPs or other ECM-related proteases, thereby influencing tumor cell behavior. Targeting the integrin signaling pathway can regulate the interactions between cells and the ECM in tumor cells. As a key transcription factor under hypoxic conditions, HIF-1α plays an important role in regulating tumor cell adaptation and angiogenesis, making it a potential therapeutic target. The targets mentioned above have been extensively studied in breast cancer treatment and show significant clinical application potential.

In conclusion, the review summarizes existing research; however, there are still many limitations that require further experimental and clinical studies to validate and expand the insights into these mechanisms. It will provide a theoretical foundation for the precision treatment of breast cancer in the future and promote the development of novel therapeutic strategies, ultimately improving patient outcomes and prognosis.

## Data Availability

The data used in the review are publicly available from several online databases. Specifically, we utilized data from the MatrisomeDB database, the UALCAN platform, and the GEO dataset GSE224147. All these datasets are freely accessible, allowing for independent verification and further analysis. Researchers interested in the datasets can access them directly through the following links: MatrisomeDB: https://matrisomedb.org/, accessed on 30 July 2024; UALCAN: https://ualcan.path.uab.edu/, accessed on 2 August 2024; GEO Dataset GSE224147: https://www.ncbi.nlm.nih.gov/geo/query/acc.cgi?acc=GSE224147, accessed on 2 August 2024.
